# A randomised control trial assessing the impact of an investment based intervention on weight-loss, beliefs and behaviour after bariatric surgery: study protocol

**DOI:** 10.1186/s40608-015-0048-2

**Published:** 2015-03-21

**Authors:** Amelia Hollywood, Jane Ogden, Majid Hashemi

**Affiliations:** Department of Psychology, University of Surrey, Guildford, GU2 7XH UK; University College London Hospital, GI Services, Ground Floor West, 250 Euston Road, London, NW1 2PG UK

**Keywords:** Bariatric surgery, Weight loss, Obesity, Behavioural intervention

## Abstract

**Background:**

Although obesity surgery is currently the most effective method for achieving weight loss, not all patients lose the desired amount of weight and some show weight regain. Previous research shows that successful weight loss may be associated with the amount of investment the patient feels that they have made in their operation. For example, those who feel that it has taken more time and effort to organise, has cost more money, has been more disruptive to their lives and has caused pain are more likely to lose weight after their operation. Therefore, it seems as if the greater the sense of investment, the greater the motivation to make the operation a success. The present study aims to build on these findings by encouraging weight loss surgery patients to focus on the investment they have made, thus making their investment more salient to them and a means to improve weight loss outcomes.

**Methods:**

The study involves an open randomised parallel group control trial with patients allocated either to the control or investment intervention group. Using third party blinded randomization, half the patients will be asked to rate and describe the investment they have made in their operation just before surgery then 3 and 6 months after surgery. All patients will record their weight, beliefs about food, intentions to change and actual eating and exercise behaviour at baseline then 3, 6 and 12 months follow up. Patients will be recruited from the bariatric surgery pre-assessment clinic at University College Hospital, London. The primary outcome is to explore the impact of the investment based intervention on patient’s weight and BMI, with secondary outcomes of patients’ beliefs about foods, behavioural intentions and diet and exercise behaviours.

**Discussion:**

It is predicted that the investment intervention will improve excess weight loss post-surgery, together with beliefs about food, intentions to change and actual change in diet and exercise behaviour. This has cost implications for the NHS and other healthcare providers as improved effectiveness of bariatric surgery reduces the health costs of obese patients in the longer term and this simple, easy to administer and low cost intervention could become routine practice for bariatric patients.

**Trial registration:**

ClinicalTrials.gov identifier NCT02045628; December 2, 2013.

## Background

Obesity is caused by people consuming more energy than they expend and is associated with reduced life expectancy and many serious conditions including heart disease, stroke, diabetes, cancer, gallstones, fatty liver disease and sleep apnoea [1,2]. Currently almost two-thirds of UK adults are either overweight or obese with overall costs to society forecast to reach £50 billion per year by 2050 on current trends [2].

Although the most common form of obesity management emphasizes changes in diet and exercise, research indicates that weight loss surgery (WLS) is most effective and this approach is currently recommended for those whose BMI is greater than 40 kg/m2 (or 35 kg/m2 with comorbidities) [3]. A systematic review of WLS [4] concluded that the mean percentage excess weight loss (EWL) for the Roux-en-Y gastric bypass was 67% and for the gastric band was 42% at one year. WLS has also been shown to result in improvements in a number of other patient outcomes including quality of life, mood, subjective health status and perceptions of eating control [5,6].

Although WLS is currently one of the most frequently performed procedures in the US and Europe [7], questions have been raised about the long-term durability of weight loss as research indicates that a substantial proportion of individuals begin to regain lost weight over time [5,8-10]. In response to growing evidence that WLS does not work for everyone research has attempted to understand this variability. In particular, research has explored the mechanisms involved in successful and failed bariatric surgery to highlight how effectiveness could be improved [11,12]. The results indicate that less successful surgery is associated with feeling unprepared for the changes required after surgery, reporting being unsupported in the time following surgery and a sense that although surgery fixes their body, psychological issues relating to dietary control, self-esteem, coping and emotional eating remain neglected. These studies also explored successful weight loss following surgery and reported a role for a reduction in hunger, a decrease in the preoccupation with food and sense of improved control. Such research also highlights a central role for investment and this is supported by other studies using medical and behavioural interventions. For example, bariatric patients reported that success was associated with a feeling that they had made an investment in their operation in terms of factors such as time of work, financial cost, disruption to family and social life, the pain of the surgery and the process of recovery. As one participant said after surgery ‘the amount of pain, the operations performed. Don’t want to do any damage, don’t particularly want the stomach to enlarge anymore which it can do’. Similarly, research indicates that obesity medication may work through the greater investment needed for adherence due to unpleasant side effects [13]. Further, success from behavioural interventions is associated with perceptions of greater investment in attempts to lose weight such as joining self-help groups or attending organised slimming clubs. From this perspective greater investment into weight loss attempts increases the chances of success by motivating the individual to make the most of the efforts they have made so far and encourages them to maximise the consequences of these efforts.

Perceived investment therefore motivates change across all areas of obesity management. This perspective finds reflection in the body of work on the effect of financial incentives on behaviour change with greater costs either facilitating positive behaviours or deterring unhealthy behaviours [14]. It is also parallel to research on the placebo effect which illustrates that placebos which involved greater investment (i.e. bigger pills, expensive treatments, painful interventions, lengthy consultations, greater distances travelled) are more effective that those which are easier to take part in [15]. In line with this approach, a recent pilot study explored the impact of manipulating perceived investment in patients just after they had had bariatric surgery [16]. Patients (n = 98) were recruited from an online support group for bariatric patients and were randomly allocated to either the control or intervention group. Those in the intervention group then completed a simple series of carefully framed questions designed to encourage them to consider the investment they had already made in having their operation. For example, they were asked to rate the procedure in terms of their pain experienced, the time spent organising the operation, the disruption to their family and social life, the time needed to recover and the impact of the recovery process on their family and work. This approach was inspired by recent research exploring the ‘mere measurement’ effect which illustrates that simply completing framed questions can change the ways in which people think and behave [17,18]. The results from this pilot study indicated that the investment intervention resulted in an immediate change in participants’ beliefs about food and their intentions to change their diet and exercise behaviour. By three months follow up the investment group reported losing 7 kg more than the control group (a significant difference). This pilot study was small scale and involved patients post-surgery only. In addition, follow up data was only collected in the short term and attrition was high by this time point (probably due to the online nature of the study). The present study aims to develop this investment based intervention using a larger sample, with a longer follow up and involving assessments at both pre and post-surgery in order to generate a proper baseline. Bariatric surgery is therefore the treatment of choice for obese patients. Not all patients, however, lose the desired amount of weight. Research highlights a role for investment which lends itself to a low cost, easy to administer intervention that may promote successful weight loss in the longer term. Therefore the aim of this study is to evaluate the impact of an investment based intervention compared to usual care on patient weight loss, beliefs and behaviours after bariatric surgery.

## Methods/Design

### Design

The study will involve an open randomised parallel group control trial with patients allocated either to the control or investment intervention group. All participants will complete measures of their beliefs about food, diet and exercise behaviour, intentions to change their behaviour and weight at baseline (2 weeks pre surgery) and at 3, 6 and 12 months follow up. Those in the investment group will complete carefully framed questions designed to raise the salience of the investment they have made in their procedure at baseline the 3 and 6 months after surgery. The content of this intervention will be tailored to the recent experiences of the patient (i.e. pre or post-surgery). This study has received favourable ethical opinions from the Bloomsbury Research Ethics Committee, National Research Ethics Service UK and the University of Surrey Ethics Committee. The structure and reporting of this trial will be guided by the CONSORT statement for clinical trials [19].

### Piloting

A pilot study has already been completed which illustrates that a low cost, easy to administer investment based intervention can change beliefs about food and improve weight loss in bariatric patients [16]. This was based upon previous research and is grounded in research on the mere measurement effect, the impact of incentives and the placebo effect. The present study is an extension of this work using a larger sample, pre and post-surgery interventions and measurements and a longer term follow up.

### Sample

University College Hospital (UCH) in London, UK, offers a NHS based standardised bariatric service for obese patients with a BMI over 40 (or 35 with serious co morbidities). Patients will be recruited if they have been approved for surgery and are attending the hospitals bariatric surgery pre-assessment clinic.

### Inclusion/exclusion criteria

Patients will be included if they consent, are aged 18 or over, have attended the bariatric clinic at UCH, been accepted for surgery and have funding in place (i.e. the CCG has agreed to pay for their surgery). Recruitment will take place over a 14 month period.

### Power calculation

The effectiveness of the proposed investment based intervention has been preliminarily examined in the pilot study conducted by Husted and Ogden [16]. The study showed that with only 98 participants (48 in intervention and 50 in the control groups) there was a significant effect of the intervention on weight loss by 3 months follow up with a mean difference of 7 kg between the groups. This resulted in a small / medium effect size (d = 0.3). The present study aims to assess outcomes up to 12 months thus raising the risk of attrition over time. In addition, the study will have a baseline measure of weight pre surgery rather than after surgery. Therefore, assuming an attrition rate of 40% by 12 months with an alpha of 0.05 and a beta of 0.8 we propose to invite 200 participants (100 in each condition) to ensure that data from at least 120 participants is collected at the 12 months follow up (60 in each group). This should provide statistical power to detect a small to medium difference in weight (controlling for baseline weight) by 12 months follow up.

### Procedure

Two weeks before their operation patients attend the bariatric clinic for routine pre-operative tests. At this point all patients will see the researcher who will explain the trial, obtain consent and randomly allocate the patient to either the control or the investment based weight loss intervention condition.

### Randomisation

Once a patient is consented the researcher will use the third party blinded randomization process provided by the clinical trial unit at the University of Surrey to allocate them to either the investment based intervention condition or the control group, using random number tables according to surgery type.

### Control group

Those allocated to the control group will receive usual care and complete a questionnaire at baseline then 3, 6 and 12 months after bariatric surgery.

### Investment intervention group

Those allocated to the investment based intervention will complete the investment based intervention at baseline (2 weeks pre surgery) then 3 and 6 months post-operatively. This intervention is based on the pilot study [16] and involves participants rating questions relating to the investment they have made in having bariatric surgery as a means to raise the salience of their investment. In particular, the questions will encourage patients to consider the ways in which the surgery has impacted upon them in terms of financial, social, personal and physical costs and focuses on factors such as pain, disruption to their family, social and work lives and financial burden. The investment intervention will be tailored to the recent experiences of the participants. To this end the questions and responses are framed in such a way as to emphasise investment in the process of bariatric surgery in order to optimise the outcomes of the procedure. They will also complete a questionnaire at baseline then 3, 6 and 12 months after surgery.

### Primary outcome measures

BMI and weight: Patients’ weight will be obtained in the clinic to provide the primary baseline and endpoint measure of the trial. This will be collected preoperatively 2 weeks before surgery, immediately after surgery and postoperatively at 3, 6 and 12 months follow up. In addition baseline measures of age, sex, height, type of surgery, educational level, and ethnicity will also be taken.

### Secondary outcome measures

Beliefs about food; including hedonic wanting using the Power of Food Scale (PFS) [20], hedonic liking relating to food palatability preference using written representations of food types replicating examples suggested in previous studies [20,21]. Behavioural intentions including patients’ intentions to eat foods high in fat and sugar content and diet and exercise behaviour using measures of snack and meals intake that have been used extensively in previous research [22,23].

### Data analysis

The data will be analysed to explore the impact of the investment based weight loss intervention on patient’s weight and BMI controlling for baseline measures using ANCOVA. Furthermore the impact of the investment intervention will be explored with regard to patients’ beliefs about foods, behavioural intentions and diet and exercise behaviours using repeated measures ANOVA. Finally the data will be analysed to assess the role of changes in beliefs about food, behavioural intentions and behaviour in predicting weight loss by follow up using mediation analysis. Any baseline differences will be included as covariates in the analysis where necessary.

## Discussion

Obesity is a risk factor for illnesses such as heart disease, diabetes and cancer. If effective, obesity surgery improves a patient’s health and reduces their need for NHS care. If unsuccessful then the costs include not only subsequent NHS costs due to these other illnesses but also the costs of the unsuccessful operation and the emotional cost to the patient. The investment based intervention aims to help improve the effectiveness of surgery which in the longer term is likely to be cost effective. This research is based upon a small scale pilot study that showed that a simple investment based intervention which encouraged patients to consider their personal investment in having weight loss surgery, improved weight loss and if this is reproduced in the current larger scale study then such a simple, easy to administer and low cost intervention could become routine practice for bariatric patients.

## Abbreviations

WLS: Weight loss surgery
EWL: Excess weight loss
UCH: University College Hospital
CCG: Clinical Commissioning Group
UK: United Kingdom
NHS: National Health Service
